# Lightweight structure-aware explanations for rule-based models: application to radiotherapy risk communication in breast cancer

**DOI:** 10.3389/fdgth.2026.1925421

**Published:** 2026-09-14

**Authors:** Alessandro Umbrico, Guido Bologna, Luca Coraci, Francesca Fracasso, Silvia Gola, Gabriella Cortellessa

**Affiliations:** 1 National Research Council of Italy, Institute of Cognitive Sciences and Technologies, CNR-ISTC, Rome, Italy; 2 University of Applied Sciences and Art of Western Switzerland, Geneva, Switzerland

**Keywords:** attribution-based analysis, decision support system, explainable artificial intelligence, information retrieval, rule-based predictive models, TF-IDF

## Abstract

**Objective:**

Rule-based models are often considered inherently interpretable because their explicit logical structure allows users to trace predictions back to human-readable rules. However, in complex domains such as healthcare, multiple rules involving numerous attributes may be activated simultaneously, making it difficult for end users to identify the most influential factors behind a prediction. This can limit practical transparency, particularly in clinical shared decision-making, where clear and trustworthy explanations are essential. An example is communicating the risk of side effects following radiotherapy for breast cancer, where understanding the determinants of estimated risk may support informed treatment choices.

**Method:**

We propose a lightweight, structure-aware attribution method for rule-based models inspired by information retrieval techniques. The approach represents the activated rules associated with a prediction as a document-like structure and applies a TF-IDF-inspired weighting scheme to estimate the relevance of individual input attributes. Unlike perturbation-based methods such as SHAP and LIME, the proposed approach operates directly on the internal model structure, without requiring input perturbations, surrogate models, repeated evaluations, or access to training data. This preserves the explanatory signal encoded in the rules while reducing computational complexity and execution time. The method is evaluated through a technical comparison with SHAP and LIME and a user-centered study involving potential end users.

**Results and Conclusions:**

Experimental results show moderate agreement between the TF-IDF-inspired ranking and established attribution methods, identifying attributes that are behaviorally informative with respect to the implemented predictor. Faithfulness analysis shows that direct rule frequency achieves higher perturbation faithfulness, highlighting a trade-off introduced by the proposed weighting scheme. Complementarily, the user study indicates that the complete explanation pipeline, combining relevance weighting, semantic aggregation, and user-oriented presentation, is perceived as more interpretable and useful than raw rule-based explanations. These findings suggest that information-retrieval-inspired weighting can provide a lightweight mechanism for organizing rule evidence, while semantic abstraction and user-oriented presentation can facilitate its communication to end users.

## 1 Introduction

Artificial Intelligence (AI) technologies have shown impressive computational capabilities that can positively impact decision-making processes. Although effective, the black-box nature of data-driven AI systems hampers their diffusion and acceptance in the real-world, especially in high-stakes decision-making domains such as healthcare. Adopting AI in clinical practice can offer potential advantages (1), such as augmenting clinicians’ work in the diagnostic phase or providing valuable personalized treatment recommendations. Nevertheless, the lack of transparency is one of the main barriers that prevent the regular adoption of such systems (2). Since clinicians must be confident in their delicate role, AI systems should be trustworthy, and Explainable AI (XAI) is paramount to their effective development (3). Explainability is closely related to *understanding*, which involves forming a mental model of what is being observed. Explaining or interpreting can be described as the process of presenting the causes behind observed phenomena in a clear and comprehensible way, using linguistic descriptions to outline their logical and causal relationships (4, 5). In the health domain, it has been argued that clinicians may feel uncomfortable with recommendations coming from black-box predictive models. AI outcomes should be explainable so that clinicians can understand the underlying (hidden) *rationale* leading to the results (6). Explaining the rationale behind AI-based predictions is crucial to justify the resulting decision-making processes (7).

Within XAI, explainability, linked to interpretability, aims to create insights into how and why AI models produce predictions. No agreement has been reached on the definition of “explainability” or “interpretability”. The work (8) proposes a distinction between the two terms. Explainability provides a target audience with insight that meets explanation needs. Interpretability is the degree to which the information provided makes sense for the target audience (8). A similar distinction is discussed in Montavon et al. (9), where an explanation is understood as a collection of features of the interpretable domain that contributed to a specific example in making a decision. An interpretation is defined instead as the mapping of an abstract concept into a domain that a human expert can perceive and understand. Despite the significant effort made in XAI, the added value of explainable AI methods remains to be proven in practice (3), and the developed solutions should be justified by their reliability and validated performance (2). Such observations outline the central role of *user-centered XAI* and the need to *frame* interpretations according to users’ expertise, mental models, and needs. Explainable AI models do not automatically imply the *understandability* of their outcomes to all stakeholders. It is necessary to consider the explanation needs and expertise of specific users and communicate the results accordingly. To this aim, it is important to rely on conceptual frameworks allowing designers and researchers to assess the quality of XAI from multiple perspectives e.g., understandability, interpretability, usefulness) (10).

Within the Horizon Europe project PRE-ACT^1^ (11, 12), XAI algorithms have been designed to predict, interpret, and communicate the individual *risk* of developing arm lymphedema after radiotherapy treatments of breast cancer. Arm lymphedema is a side effect that might significantly impact the quality of life of patients. The ability to provide appropriate predictions about the risk of arm lymphedema enables more personalized treatment planning and the provision of lifestyle advice. Within PRE-ACT, research efforts investigate the design of an additional layer to augment the interpretability of an explainable AI prediction model (13), under the hypothesis that such a layer would improve user understanding of AI predictions. Thus, the current work introduces and evaluates an XAI approach designed to improve the interpretability of a rule-based model (13).

A key challenge concerns the clinical interpretability of predictions going beyond the “technical justification” of the underlying opaque learning mechanisms. In this regard, the use of “explainable AI models” such as van der Waa et al. (14), although effective, is far from being fully understood by users without a proper technical background in AI and interested in domain-level (clinical) insights. The proposed method relies on Information Retrieval (IR) to compute the relevance of input attributes by analyzing their occurrence and distribution across the rules activated for a given prediction. It aggregates input variables (i.e., model attributes) into semantically coherent *factors* and computes *relative scores* characterizing their *contribution* to individual predictions. Semantic aggregation provides the framework with interpretation flexibility suitable for tailoring explanations to users’ expertise and to the desired level of detail concerning the model attributes. The resulting factor-level representation is intended to preserve a structured connection with the evidence encoded in the activated rules while presenting such evidence at a granularity more suitable for clinicians and patients. Importantly, the TF-IDF-inspired weighting determines the relevance assigned to the contributing factors, whereas semantic aggregation and visualization determine how this information is organized and communicated to end users. The user-centered evaluation therefore assesses the resulting integrated explanation pipeline rather than the contribution of the weighting mechanism in isolation.

## 2 Motivations and positioning with the state of the art

The work addresses the problem of improving the interpretability of a rule-based prediction model. Although rule-based models expose the logical conditions supporting each prediction in the shape of *decision trees*, the resulting explanations are not always readily understandable to clinicians and patients, particularly when multiple rules jointly contribute to the same prediction or when the same clinical variable appears repeatedly across different logical conditions. An influential perspective on XAI argues that, especially in high-stakes domains such as healthcare, the preferred solution is not to explain opaque predictive models but rather to employ inherently interpretable models whenever possible (15). Rather than relying on post-hoc approximations, this paradigm advocates transparent decision processes whose reasoning can be directly inspected and verified. Similarly, Molnar et al. (16) distinguishes between intrinsically interpretable models and post-hoc explanation techniques, emphasizing that explanation quality depends not only on *fidelity* to the predictive model but also on *human comprehensibility*. These observations provide the primary motivation for the present work. Instead of replacing an opaque predictor with post-hoc explanations, we consider a rule-based predictive model and investigate how its explanations can be semantically reorganized to improve communication with different categories of users.

A large body of XAI research has focused on explaining complex predictive models through feature-attribution techniques and *surrogate models*. Surrogate-based approaches approximate the behavior of a reference model while providing improved interpretability (17, 18). However, these methods typically require access to training data, additional optimization procedures, and retraining whenever the predictive model changes. Among model-agnostic explanation techniques, SHAP (19) and LIME (20) estimate *feature relevance* through perturbations of the input space and repeated evaluations of the predictive model. Recent applications have successfully exploited these techniques to improve transparency in healthcare prediction systems (21, 22). Nevertheless, their underlying assumptions are not naturally aligned with symbolic rule-based systems, where predictions arise from combinations of logical conditions and the same variable may contribute multiple times across different activated rules.

Beyond feature-level explanations, recent research has progressively shifted toward concept-level explanations that express model behavior using higher-level semantic concepts. Kim et al. (23) introduced Testing with Concept Activation Vectors (TCAV), which quantifies the influence of user-defined concepts on neural network predictions by analyzing latent activation spaces. Similarly, Concept Bottleneck Models (24) explicitly introduce semantic concepts as intermediate representations driving the final prediction, enabling users to inspect and intervene in the reasoning process. Although these approaches successfully bridge low-level model representations and human-understandable concepts, they remain tightly coupled with neural architectures and require either latent representations, predefined concept annotations, or architectural modifications. In contrast, our work addresses a complementary problem by deriving semantic concepts directly from activated symbolic rules, making the framework applicable to already deployed rule-based predictive systems without retraining or access to internal latent representations.

The evaluation of explainability has also attracted multidisciplinary efforts pushing the human perspective into the explanation process. Mohseni et al. (25) considers explainability as a complete human-centered process, advocating the joint evaluation of explanation correctness, usability, trust, and human performance. Likewise, Vilone and Longo (26) provide a comprehensive survey of explainability notions and evaluation methodologies, emphasizing that no single metric is sufficient to characterize explanation quality and highlighting the complementary role of computational and human-centered evaluations. Following these recommendations, the proposed framework is evaluated through both quantitative comparison with established attribution methods and a user study assessing the comprehensibility and perceived usefulness of the generated explanations. Recent work has also started investigating explanations of prediction reliability rather than predictions themselves. For example, Brandsæter and Glad (27) employ Shapley-value analysis to explain prediction accuracy in hindsight by analyzing the contribution of training samples. While complementary to our objectives, these approaches remain fundamentally based on perturbation analysis and feature attribution, without explicitly exploiting the logical structure of rule-based predictors.

Existing XAI research has primarily focused either on explaining opaque predictive models through feature-attribution techniques or on designing inherently interpretable predictive models. Little attention has been devoted to enriching the explanations generated by symbolic rule-based systems through higher-level semantic abstractions tailored to different categories of users. The framework proposed in this paper addresses this gap by introducing a lightweight, structure-aware semantic explanation layer that analyzes the activated rules of the predictive model, estimates the structural relevance of the contributing factors, and reorganizes variable-level explanations into clinically meaningful concepts. Rather than replacing existing attribution methods, the proposed framework complements them by reorganizing model evidence into representations intended to support human interpretability, while preserving a direct connection with the structural reasoning encoded by the predictive model.

## 3 Method

The proposed approach occupies a distinctive position between intrinsically interpretable models and post-hoc attribution techniques. It preserves a direct connection with the structure of the rule-based explanations while introducing a quantitative, flexible, and computationally efficient measure of feature relevance. This combination of properties makes it particularly suitable for real-world scenarios where interpretability, scalability, and responsiveness are required, such as user-centered interactive decision-support systems. The proposed explanation framework therefore comprises three conceptually distinct components: (i) a structure-aware weighting mechanism that estimates relevance from the activated rules; (ii) a semantic aggregation layer mapping model attributes to clinically meaningful factors; and (iii) a presentation layer communicating the resulting factor-level explanation to end users. The following evaluation considers these components from complementary perspectives: the technical experiments assess the properties of the weighting mechanism, whereas the user study evaluates the integrated user-facing explanation.

The EU Project PRE-ACT^2^ aims to develop AI-based prediction capabilities to calculate the risk of arm lymphedema for patients and to design appropriate communication strategies to deal with or mitigate the (predicted) risk (11, 12). Also, it investigates explanation and communication techniques that are suitable for effectively interpreting arm lymphedema risk in breast cancer for patients who should be treated with loco-regional radiotherapy (11, 12). PRE-ACT, therefore, aims to address several of the open challenges concerning explainability for “clinicians” (2, 28).

Predictions are expressed as logical conjunctions among numeric conditions on the model’s attributes (13). Hereafter, more information on the XAI model adopted in this context to predict arm lymphedema after nodal irradiation. The Discretized Interpretable Multi-Layer Perceptron (DIMLP) is a neural network architecture specifically designed to combine the predictive performance of a multilayer perceptron (MLP) with the ability to extract human-readable symbolic rules. It was introduced as an alternative to standard MLPs (29). Typically, these models use smooth sigmoidal activations in its hidden neurons, producing complex nonlinear decision boundaries that are difficult to convert into symbolic rules. In contrast, DIMLP replaces the sigmoid activations of the first hidden layer with staircase functions and restricts each hidden neuron to be connected to a single input variable and the bias unit. This architecture discretizes each input dimension independently into a set of intervals. The resulting partition of the input space forms hyper-rectangular regions that can be directly translated into propositional rules expressed as conjunctions of conditions on input attributes, thereby facilitating model interpretation and rule extraction (30). Rule extraction is performed by Fidex, which is a local, greedy algorithm that generates propositional rules to explain individual sample classifications by DIMLP networks and other models (13, 31). It exploits the precisely located axis-parallel discriminative hyperplanes inherent to DIMLP architectures to build interpretable rules with high fidelity, defined as the proportion of samples for which the rule predictions are consistent with those of the original model. Fidex determines a propositional rule for a given sample by iteratively selecting hyperplanes that maximize fidelity. At each step, the algorithm identifies the hyperplane that increases fidelity the most, up to a user-specified number of steps. The candidate hyperplanes are drawn from a finite list computed from the first hidden layer’s weights for each input variable (31).

FidexGlo extends Fidex to global rule extraction by calling Fidex once per training sample, then applying a covering heuristic to prune the ruleset (13, 31). The heuristic ranks rules in descending order by the number of samples covered and selects rules until all training samples are covered. FidexGlo demonstrated competitive results on benchmark classification problems when applied to DIMLP ensembles and support vector machines (13, 31). Both Fidex and FidexGlo are publicly available as open-source tools.^3^ Bagging, or bootstrap aggregating, was used in this work to train DIMLP ensembles. Bagging creates multiple subsets of training data by randomly sampling with replacement, trains a separate model on each subset, and averages or votes on their predictions. The technique is particularly effective for complex, unstable learners such as neural networks, reducing variance without increasing bias (32–34). A bootstrap sample typically contains about 67% of the original data samples due to replacement sampling.

DIMLP networks are bagged by training multiple DIMLP instances on bootstrap replicates, then aggregating their predictions. The resulting ensemble is treated as a single DIMLP with an additional hidden layer that averages all individual network responses. This architectural equivalence enables rule extraction from the entire ensemble using rule extraction algorithms like FidexGlo. For the arm lymphedema prediction task, experiments were performed using ten repetitions of 10-fold cross-validation. DIMLP ensembles were composed of 30 networks, each containing 79 input neurons, a first hidden layer of 79 neurons with 50-step staircase activation functions, a second hidden layer of 10 neurons, and two output neurons representing the target classes. The dataset included 4,177 samples, of which 259 were positive cases, corresponding to a 6.2% incidence rate. The prediction outcome was the occurrence of arm lymphedema after breast cancer treatment and regional nodal radiotherapy. The definition of arm lymphedema is ≥5% increased arm circumference measured 15 cm proximal and/or 10 cm distal to the olecranon of the treated side from baseline, compared to the contralateral side circumference and to its baseline value.

The DIMLP ensemble and rule-extraction pipeline were implemented using version 1.0.^4^ of the publicly available DIMLP/Fidex software distribution. During the repeated cross-validation experiments of the original DIMLP model, the random-number generator was initialized using process identifiers obtained from the Linux execution environment through getpid(). Consequently, the original training procedure did not rely on a single manually fixed numerical seed. Following standard DIMLP design principles, the size of the first hidden layer was set equal to the number of input features. The number of neurons in the second hidden layer was selected such that the total number of trainable weights remained below the number of training samples, an empirical rule intended to reduce overfitting. A second strategy was employed to reduce overfitting: early stopping based on out-of-bag samples. Approximately 33% of the training samples are not selected during the bootstrap sampling process and therefore constitute the out-of-bag set. Training was halted when performance on these out-of-bag samples ceased to improve, thereby helping to prevent overfitting and enhancing the model’s generalization ability. Two predictive models were initially evaluated, namely Random Forests and an ensemble of DIMLPs. Model selection was based on ten repetitions of stratified 10-fold cross-validation. The DIMLP ensemble achieved slightly better discrimination and calibration than Random Forests (mean AUC 0.832±0.003 vs. 0.824±0.003), together with calibration slopes closer to the ideal value of one and low calibration bias. Consequently, the DIMLP ensemble was selected as the predictive model employed throughout this study. A ruleset containing 207 rules with an average of 3.5 conditions per rule was generated by FidexGlo to represent all data samples.

The AI model was trained on a large dataset extracted from data derived from three different cohorts of previous studies: (i) REQUITE (2057 patients, data collected between 2014 and 2019) (35); (ii) CANTO-RT (3918 patients, data collected between 2012 and 2022) (36); (iii) the UNITRAD HypoG-01 trial (1265 patients, data collected between 2016 and 2022) (37).

From the three cohorts, 4,177 samples satisfying the inclusion criteria defined for the PRE-ACT clinical study were retained. Eligible patients were women ≥18 years with unilateral invasive breast cancer (cT1-4, cN0-3, M0) after mastectomy or breast-conserving surgery with negative margins, regardless of receptor status or malignancy grade, and with an indication for regional nodal radiotherapy. Missing or unknown categorical information was explicitly represented in the model input through dedicated variables (e.g., *_UNKNOWN), rather than being implicitly treated as the absence of the corresponding clinical condition. The original clinical variables were transformed into the input representation required by the predictive model. Data were normalised; categorical inputs with a missing value were coded by a supplemental vector component, while continuos/discrete values of missing variables were replaced by their median values. Categorical variables were represented through dedicated indicator attributes, while derived clinical variables, such as BMI, were computed from the corresponding source measurements. The resulting DIMLP input space comprised 79 model attributes.

In the given context, the proposed approach bridges intrinsically interpretable models and post-hoc attribution methods by preserving a direct connection with the structure of the rule-based explanations while providing a quantitative, flexible, and computationally efficient measure of feature relevance. These characteristics make it well-suited to real-world decision-support applications that require interpretability, scalability, and responsiveness. Additionally, semantic clustering groups model attributes into meaningful factors, enabling explanations at different levels of abstraction. By adjusting the clustering granularity, the interpretation can be tailored to the needs and expertise of different users. Given the mapping between attributes and factors, the designed XAI service analyzes the activated rules and computes the structural relevance of each factor to the prediction. Specifically, the TF-IDF-inspired metrics determine: (i) the subset of factors represented in the activated rules; and (ii) the relative relevance assigned to each active factor according to its occurrence and distribution across those rules.

### 3.1 Beyond rule-based explanations

The use of IR metrics is largely inspired by the fact that the rule-based model (13) makes predictions by activating an arbitrary number of rules for a given input (i.e., patient data). Each rule consists of a conjunction of numerical constraints over input variables that determine the predicted risk class. An attribute (or associated factor) may be absent from the activated rules, indicating that it does not contribute to the structural explanation considered for that prediction, or may appear multiple times across different rules.

The underlying assumption is that the more frequently a set of attributes occurs in the preconditions of the activated rules, the greater the structural relevance assigned to the associated factor. Therefore, attributes, similarly to tokens in documents, could appear zero or multiple times in one or more rules. Considering a prediction as a collection of rules, it is possible to assess the relevance of attributes (and associated factors) to a prediction in the same way IR assesses the relevance of tokens to a collection of documents. Figure 1 schematizes a mapping between IR concepts and rule-based prediction elements.

**Figure 1:**
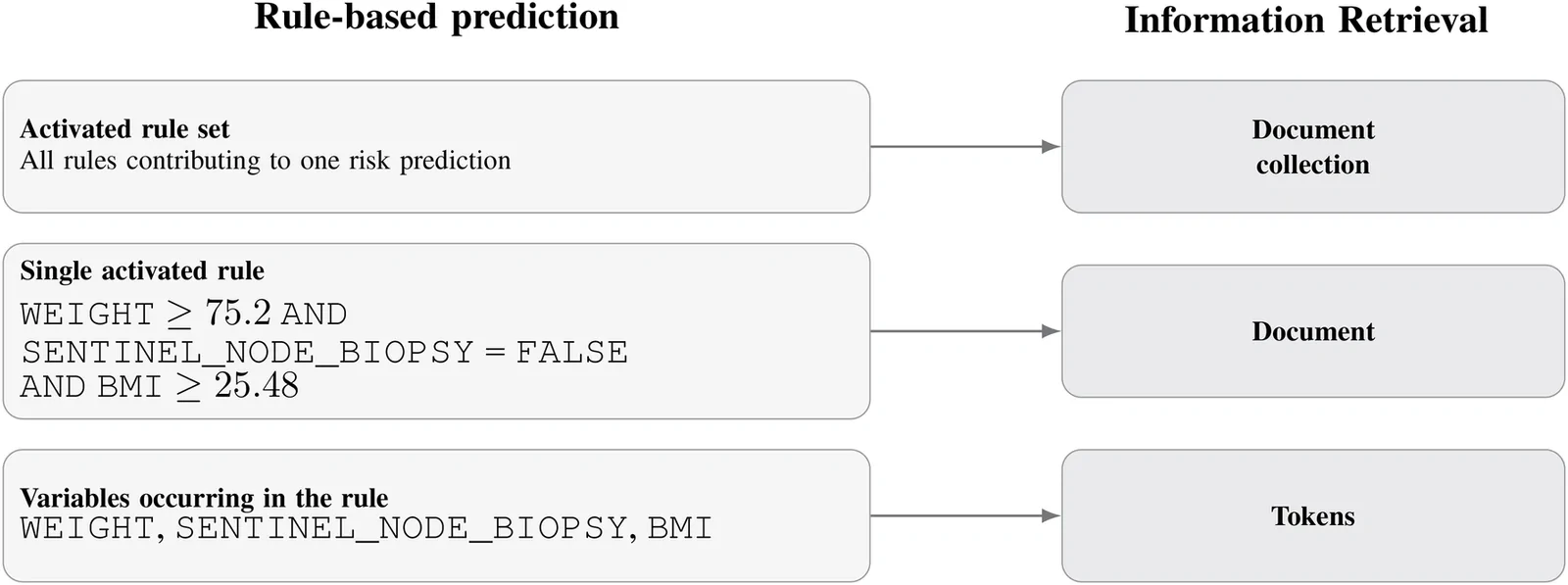
Conceptual mapping between Information Retrieval concepts for document indexing and the proposed application to rule-based explanation: (i) collection of documents—activated rules; (ii) document—single rule; (iii) document tokens—precondition variables.

Recalling IR (38), the objective of TF-IDF is to infer the amount of information conveyed by a token to classify the content of documents within a collection. The metric generally assigns higher values to tokens that are not widely used over the collection but are specific to a subset of documents. The assumption is that tokens that are not common but frequent only in some documents are more specific and better capture the content of those documents. In this context, each rule is seen as a document, and each factor as a token. Factors are counted according to the occurrence of the associated attributes in the rules’ conditions. Unlike conventional IR applications, the objective here is not to identify terms that are discriminative because they occur in only a few documents. Rather, the explanation aims to emphasize factors that recur across several rules activated for the same prediction. We therefore adapt the document frequency component so that factors occurring in a larger proportion of the activated rules receive higher scores. This adaptation intentionally reverses the intuition of the classical IDF to reflect the structural explanation requirements of the considered application. Specifically, factors whose attributes occur in the preconditions of several of the rules activated for a given patient, are supposed to *weigh* more than factors whose attributes appear in fewer preconditions and fewer rules.

### 3.2 Semantic clustering of attributes

The method supports flexible attribute clustering to personalize the explanation to the profile or expertise of target users. Clustering determines a taxonomic structure linking the attributes of the AI model to contributing factors. Figure 2 shows an excerpt of such clusterings. The nodes in the upper part are the factors, while the nodes in the lower part are attributes of the model. The edges pointing from the top (the factors) to the bottom (the attributes) describe the aggregations. Specifically, leaves are the attributes used by the model, and upper nodes are increasingly more abstract factors that aggregate semantically coherent attributes. Different levels of granularity in the taxonomical structure can support different explanation needs and adapt risk communication to different stakeholders (e.g., doctors, AI experts, patients). For example, AI experts may prefer a *technical interpretation* showing a high level of detail to assess/debug the model. They might prefer a 1:1 mapping between the factors and the attributes. Doctors would prefer a *clinical interpretation*, aggregating attributes into coherent sets without considering syntactic details of the underlying model. The approach here considers in particular a two-layer taxonomical structure mapping attributes to semantically coherent factors.

**Figure 2:**
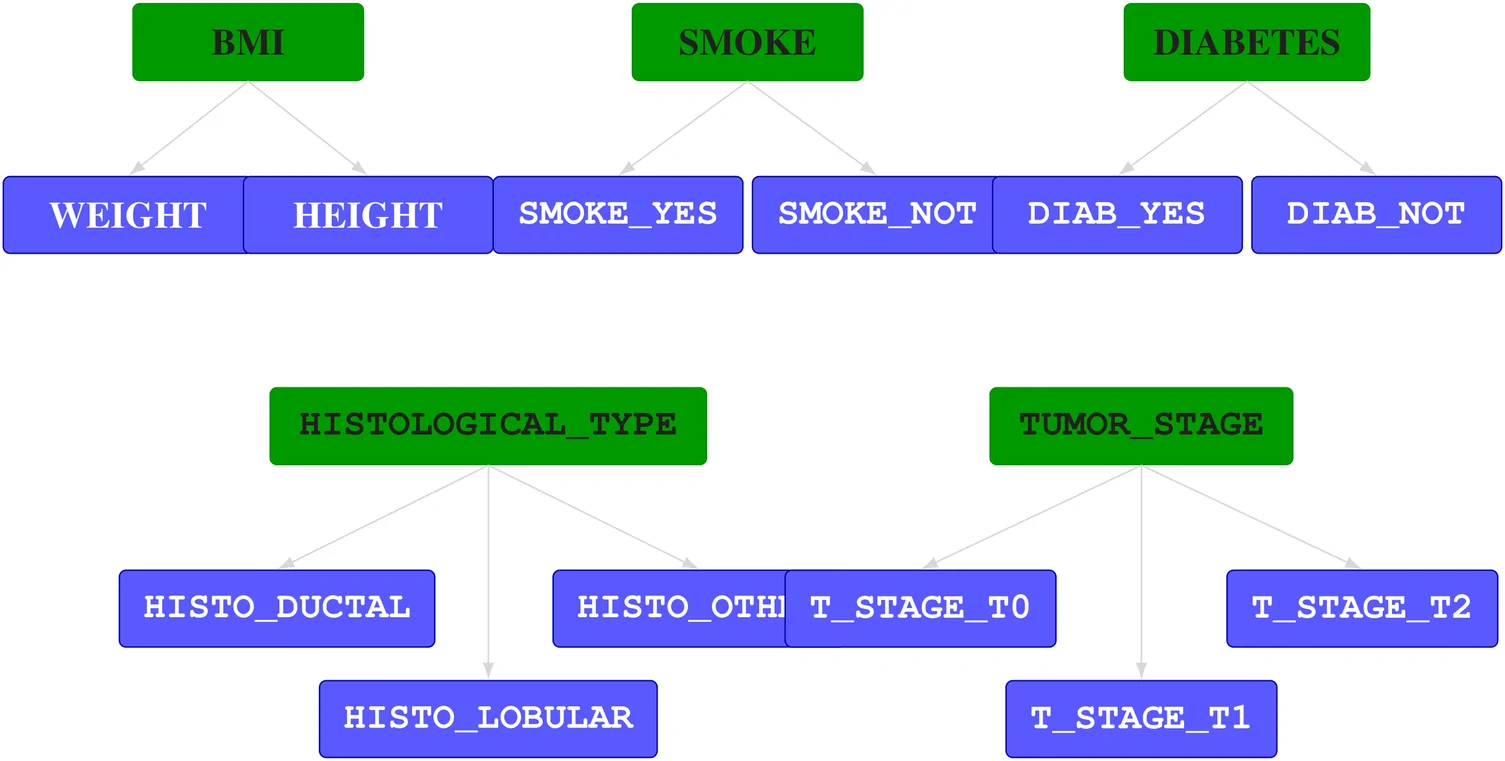
Example of the semantic aggregation adopted by the proposed explanation framework. Higher-level clinical factors are represented by green nodes, while the original predictive-model variables associated with each factor are represented by blue nodes.

The semantic taxonomy adopted in this work and within PRE-ACT was developed through an iterative co-design process involving AI researchers and the clinical partners of the project. Starting from the attributes used by the predictive model, variables were progressively grouped into clinically meaningful factors reflecting concepts commonly considered during patient assessment and treatment planning. The taxonomy was iteratively reviewed and refined until a shared consensus on the semantic organization was reached. As shown in Figure 2, each model attribute is associated with a single semantic factor, preserving an unambiguous interpretation of the attribution scores and avoiding the distribution of a variable’s contribution across multiple semantic categories. Although the taxonomy was not evaluated through an independent annotation study, it represents the shared conceptual vocabulary adopted throughout the PRE-ACT project for communicating patient characteristics and associated risk factors.

Semantic clustering groups multiple attributes into clinically meaningful factors, thereby reducing the complexity of the explanation space. Table 1 summarizes the semantic factors and the corresponding attribute mapping. The final predictive model operates on 79 input attributes. During the iterative co-design process, however, the semantic mapping dictionary was progressively extended to accommodate alternative and legacy attribute labels introduced across different versions of the prediction model. For consistency and backward compatibility, these entries were retained even when they were no longer part of the final predictive model input. The resulting dictionary therefore contains 181 attribute labels mapped onto 29 semantic factors, while only the subset corresponding to the 79 final model attributes is used by the deployed version of the predictor. For the final predictive model, semantic aggregation maps these 79 attributes onto 29 clinical factors, corresponding to an approximate 63% reduction in the dimensionality of the user-facing explanation space.

**Table 1:** Semantic factors used for explanation generation. The mapping dictionary contains 181 attribute labels accumulated across successive co-design iterations, while the final predictive model operates on 79 input attributes. Representative labels associated with each factor are shown.

Factor	# Attributes	Representative attributes
BMI	2	WEIGHT, BMI
Age	1	AGE
Height	1	HEIGHT
Smoker	10	SMOKER_YES, SMOKER_CURRENT, SMOKER_FORMER
Menopausal Status	9	PRE_MENOPAUSAL, POST_MENOPAUSAL
Diabetes	6	DIABETES_YES, DIABETES_NO
Surgery	9	MASTECTOMY, LUMPECTOMY, SEGMENTECTOMY
Axillary Nodes Dissection	2	AXILLARY_DISSECTION
Nodes Removed	2	NODES_REMOVED, NODES_EXAMINED
Nodes Involved	1	NODES_INVOLVED
Sentinel Nodes Biopsy	2	SENTINEL_NODE_BIOPSY
Primary Tumor Side	7	PRIMARY_LEFT, PRIMARY_RIGHT
Histological Type	8	DUCTAL, LOBULAR, OTHER
Tumor Size	2	TUMOUR_SIZE, PATHOLOGICAL_TUMOUR_SIZE
Clinical T Stage	22	T0, T1, T2, T3, T4
Clinical N Stage	12	N0, N1, N2, N3
Mean Heart Dose	1	MEAN_HEART_DOSE
KI67 Status	2	KI_67, KI_67_STATUS
ER Status	6	ER_STATUS_POSITIVE, ER_STATUS_NEGATIVE
PR Status	6	PR_STATUS_POSITIVE, PR_STATUS_NEGATIVE
HER2 Status	6	HER2_STATUS_POSITIVE, HER2_STATUS_NEGATIVE
Fractions Planned	2	NUMBER_OF_FRACTIONS, FRACTIONS
RT Technique	20	IMRT, VMAT, TOMOTHERAPY, 3D
Treated Breast Side	6	BREAST_RADIO_LEFT, BREAST_RADIO_RIGHT
Boost	5	BOOST, BOOST_YES, BOOST_NO
Neoadjuvant Chemo	8	NEOADJUVANT_CHEMOTHERAPY_YES, NO
Adjuvant Chemo	8	ADJUVANT_CHEMOTHERAPY_YES, NO
Baseline Arm Lymphedema	8	BASELINE_ARM_LYMPHEDEMA_YES, NO

### 3.3 Computing the impact of factors

The factor space determined by the selected clustering constitutes the “interpretation language” of the TF-IDF-inspired attribution mechanism. Algorithm 1 describes the procedure implemented to compute the relevance of factors for a given prediction. The procedure requires: (i) $R_{x}\subseteq R$, the set of rules activated by an input x; and (ii) ψ:A→F, the clustering function mapping model attributes A to semantic factors F. The procedure returns a relevance assignment $\Lambda =\{\langle f,\rho_{f}\rangle \;:\;f\in F_{x}\}$, where $F_{x}\subseteq F$ denotes the set of factors occurring in the activated rules and $\rho_{f}\in R$ represents the relevance assigned to factor f.

Algorithm 1 **Computation of factor relevance for a given prediction.**

**Require:** $\langle R_{x},\psi \rangle$

**Ensure:** $\Lambda =\{\langle f,\rho_{f}\;\rangle :f\in F_{x}\}$

 1: Λ←∅               ▹ Initialize factor relevance assignment

 2: $F_{x}\leftarrow \mathrm{factors}\;(R_{x},\psi )$               ▹ Get factors occurring in activated rules

 3: **for** $f\in F_{x}$ **do**

 4:  $R_{f}\leftarrow \mathrm{rules}\;(f,R_{x},\psi )$               ▹ Get activated rules containing f

 5:  $x_{\mathrm{idf}}\leftarrow \mathrm{adaptedFrequency}\;(f,R_{x},\psi )$               ▹ Compute inverse frequency according to Equation 5

 6:  $X_{\mathrm{tfidf}}\leftarrow \emptyset$               ▹ Initialize TF-IDF scores of f

 7:  **for** $r\in R_{f}$ **do**

 8:   $x_{\mathrm{tf}}\leftarrow \mathrm{frequency}\;(f,r,\psi )$               ▹ Compute weighted frequency according to Equation 4

 9:   $X_{\mathrm{tfidf}}\leftarrow X_{\mathrm{tfidf}}\cup \{x_{\mathrm{tf}}\cdot x_{\mathrm{idf}}\}$

 10: **end** **for**

 11:  $\rho_{f}\leftarrow \mathrm{relevance}\;(X_{\mathrm{tfidf}})$               ▹ Compute factor relevance according to Equation 7

 12:  $\Lambda \leftarrow \Lambda \cup \{\langle f,\rho_{f}\;\rangle \}$

 13: **end** **for**

 14: **return** Λ

The procedure first identifies the set $F_{x}\subseteq F$ of factors occurring in the activated rules $R_{x}$. For each factor $f\in F_{x}$, it selects the subset $R_{f}\subseteq R_{x}$ of activated rules in which f occurs. The procedure then computes the frequency of the factor within each rule by considering the occurrence of the attributes associated with f. In addition, it computes the frequency of f across the activated rule set by considering the number of rules in $R_{x}$ containing at least one attribute associated with the factor. The resulting TF-IDF-inspired scores combine these two components to characterize the structural relevance of f within the prediction. Finally, the relevance $\rho_{f}$ is obtained by considering the maximum score achieved by f over the activated rules in which it occurs. The weighting operations introduced in Algorithm 1 build upon the classical TF-IDF formulation originally developed in Information Retrieval (38), while introducing several adaptations motivated by the characteristics of rule-based explanations. Unless otherwise stated, the following equations correspond to either standard TF-IDF definitions or extensions proposed in this work. More formally, let $n_{ij}$ denote the number of attributes associated with factor i appearing in rule j, and let $d_{j}$ be the total number of attributes occurring in rule j. The local term frequency of factor i in rule j is defined below, Equation 1.

$$tf_{ij}=\frac{n_{ij}}{d_{j}}$$ (1)

Each rule is weighted according to its coverage, Equation 2. Let $c_{j}$ be the coverage of rule j and let

$$c_{\mathrm{all}}=\sum_{\;j\in J}c_{j}$$ (2)

be the cumulative coverage of all rules activated for the current prediction. The coverage weight of rule j is shown in Equation 3.

$$w_{j}=\frac{c_{j}}{c_{\mathrm{all}}}$$ (3)

The final weighted term frequency is described by Equation 4.

$$tf_{ij}=w_{j}\cdot \frac{n_{ij}}{d_{j}}$$ (4)

The equations above correspond to the standard TF-IDF weighting scheme, adapted here only through the introduction of rule coverage weights to account for the relative importance of activated rules. Let $s_{i}$ denote the number of activated rules containing factor i and let $s_{\mathrm{all}}$ denote the total number of activated rules. Since our objective is to identify factors that are recurrent across the activated rules of a prediction, the inverse document frequency is adapted to reward factors that occur in many rules as shown by Equation 5:

$$idf_{i}=100\cdot \frac{s_{i}}{s_{\mathrm{all}}}$$ (5)

According to IR principles (38), rare terms receive higher importance. In the considered scenario, the objective is slightly different, as the analysis aims to emphasize factors that consistently contribute to the prediction across multiple activated rules. Unlike the classical inverse document frequency formulation, the proposed approach intentionally modifies the document-frequency component to reward factors recurring across multiple activated rules.

The approach intentionally reverses the classical TF-IDF intuition. While traditional information retrieval seeks discriminative terms that appear in a few documents, explanation generation requires identifying factors that repeatedly support the same prediction across multiple activated rules. Consequently, factors occurring in a larger fraction of the activated rules receive higher relevance scores. The TF-IDF score of factor i in rule j is then computed according to Equation 6.

$$tfidf_{ij}=tf_{ij}\cdot idf_{i}$$ (6)

The score of a factor i is obtained by Equation 7, which considers the maximum TF-IDF value among the activated rules:

$$tfidf_{i}=\max_{r_{j}\in R_{f}}tfidf_{ij}$$ (7)

where $R_{f}\subseteq R_{x}$ denotes the subset of activated rules in which factor f occurs. We adopted the maximum operator rather than the average because a factor might strongly influence a prediction through a small subset of highly relevant rules. The use of the maximum operator constitutes a design choice of the proposed framework and differs from standard aggregation strategies typically adopted in IR.

Averaging across all activated rules would dilute localized contributions and reduce the explanation’s ability to highlight decisive factors. The maximum aggregation, therefore, captures the strongest evidence for a factor’s relevance while preserving the local nature of the explanation. Finally, the score is normalized through a logistic function in Equation 8.

$$tfidf_{i}^{*}=\frac{1}{1+e^{-tfidf_{i}}}$$ (8)

Finally, a logistic normalization is applied to map relevance scores into the interval [0,1], facilitating comparison across predictions and supporting a consistent visual representation of factor relevance. The resulting value $tfidf_{i}^{*}$ belongs to the interval [0,1] and represents the relevance of factor i within the explanation. Factors obtaining higher values are those that appear frequently across the activated rules and are associated with rules having a high relative coverage.

To summarize, the proposed weighting scheme distinguishes between the concentration of a factor within a single activated rule and its recurrence across multiple activated rules. The term-frequency component captures the relative number of attributes associated with factor (i) within rule (j), whereas the adapted document-frequency component captures the proportion of activated rules in which the factor occurs. Consequently, factors that participate in several rules supporting the prediction receive greater relevance than factors whose attributes are concentrated in a single rule. This design reflects the assumption that recurrent participation across multiple activated logical paths constitutes stronger structural support for the prediction. It is important to clarify the interpretation of the proposed attribution score. The method operates only on the rules activated by the input under analysis. Since a rule is activated only when all conditions in its antecedent are satisfied, the occurrence of a factor in an activated rule indicates that the corresponding input condition participated in the logical support of that rule. The resulting score thus measures the structural relevance of a factor within the activated rule set. It should not be interpreted as an estimate of clinical causation or as a guarantee that perturbing the factor would change the final predicted class, since multiple activated rules may provide overlapping support for the same outcome.

## 4 Results

The first part of the assessment focuses on the technical implementation of the proposed analysis, showing how the approach enriches both global and local explanations by contextualizing *factor impact* across different risk predictions. The second part of the assessment compares the proposed TF-IDF-inspired approach with standard SHAP and LIME attribution-based methods. These experiments consider a large number of realistic samples taken from the training data of the predictive model developed within PRE-ACT (13). Finally, the third part of the assessment focuses on the efficacy of the generated interpretations. It analyzes feedback from users of different fields, categorized into AI experts (e.g., AI developers, data analysts) and non-AI experts (e.g., health professionals, clinical researchers, caregivers), to evaluate the perceived interpretability and usefulness (10).^5^

### 4.1 Feasibility of global and local explanations

A global explanation analyzes the implicit knowledge encoded in all the rules of the AI module without referring to a specific input (local explanation). It enables domain experts to assess the model’s coherence with existing literature by verifying the presence of known relevant factors, while also potentially revealing unexpected or novel associations, correlations, or errors in the model (3). The proposed analysis evaluates factor relevance across the entire rule set by identifying factors with scores above average, which are considered particularly influential because they frequently appear in rule preconditions. The factor BMI obtains the highest score, making it the most relevant variable in the model, which is consistent with clinical evidence, as high BMI is a known risk factor for arm lymphedema after radiotherapy. Similarly, NODES INVOLVED and RT TECHNIQUES, representing the number of positive lymph nodes and the radiotherapy approach, respectively, also show above-average relevance, aligning with established clinical findings (40, 41).

Local explanations focus on individual patient predictions by analyzing only the subset of rules activated for a given input. Since patients have different clinical profiles, different sets of rules are triggered for each case and therefore different contributing factors. The interpretation highlights the factors most strongly represented in the activated rules supporting each patient’s prediction and shows their relative relevance according to the proposed weighting scheme. The radar charts in Figure 3 clearly show how the interpretation of local explanations evaluates the relevance of model factors differently, depending on the input features of the different patients and the rules activated within the prediction (i.e., local rules).^6^

**Figure 3:**
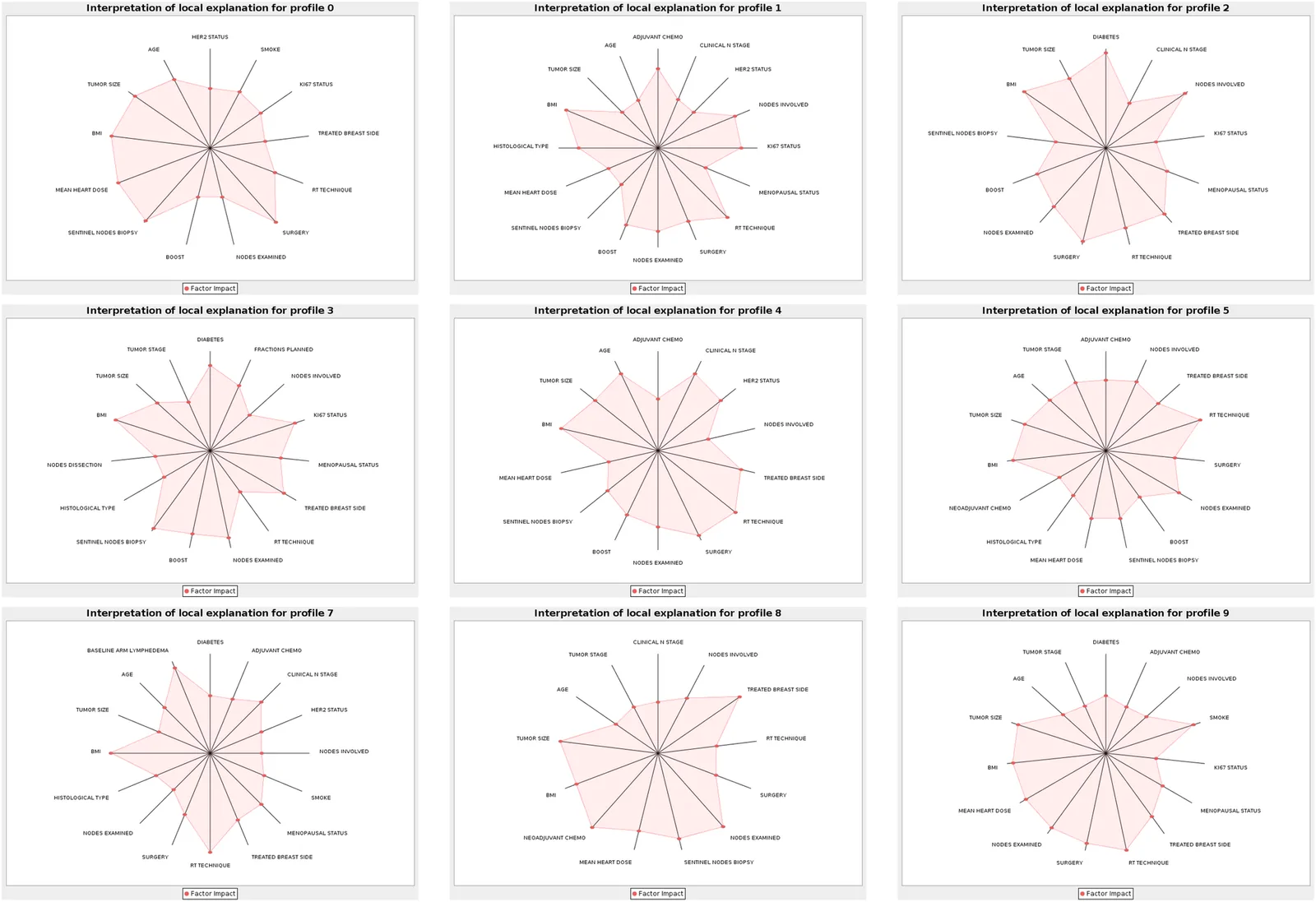
Interpretations of the local explanations generated for different patient profiles.

### 4.2 Analysis of agreement with state-of-the-art attribution methods

An additional assessment compares the TF-IDF-inspired approach with two widely adopted post-hoc explanation techniques, namely SHAP (19) and LIME (20). The objective is to evaluate the degree of agreement between the attribution patterns produced by the structure-aware method and those obtained with established, but computationally demanding, feature-attribution approaches. SHAP and LIME are used as reference methods rather than ground-truth explanations. The predictive model corresponds to the rule-based predictor developed within the PRE-ACT project (13), whose architecture, training, and predictive function have been summarized above. The analysis thus focuses on explanations derived from its activated rules. To ensure a consistent comparison, all methods are applied to the same reconstructed prediction function. Given an input instance x and the complete rule set R, the predictor first identifies the subset of rules whose antecedents are satisfied:

$$R_{x}=R(x)=\{r\in R|\text{the antecedent of }r\text{ is satisfied by }x\}.$$ (9)

Each matched rule contributes its training-confidence weight to its consequent class. If $R_{c}(x)$ is the subset of matched rules predicting class c, Equation 10 computes the operational class score as:

$$s_{c}(x)=\sum_{r\in R_{c}(x)}\mathrm{conf}(r),$$ (10)

and the normalized probability-like output results from Equation 11.

$$f_{c}(x)=\frac{s_{c}(x)}{\sum_{c^{\prime }\in \{\mathrm{NEG},\mathrm{POS}\}}s_{c^{\prime }}(x)}.$$ (11)

When no rule matches, both classes receive score 0.5. Otherwise, the predicted class is computed according to Equation 12.

$$\hat{y}(x)=\arg\;{\max|}_{c\in \{\mathrm{NEG},\mathrm{POS}\}}f_{c}(x).$$ (12)

SHAP and LIME treat f(⋅) as a black-box function. SHAP estimates feature contributions from model evaluations over perturbed coalitions, whereas LIME generates a neighborhood around x and fits a locally weighted linear surrogate. In contrast, the proposed method computes relevance directly from R(x) without perturbing the input or repeatedly evaluating the predictor. The explanation experiments were implemented in Python (v 3.10.12) using SHAP (v 0.49.1) and LIME (v 0.2.0.1). SHAP explanations were generated using Kernel SHAP (shap.KernelExplainer), with the sampling configuration described above.

The clinical input file contains 4,177 patient records described by 79 model attributes. A case was included when both a parsed local-rule explanation and a TF-IDF attribution vector were available, resulting in 3,670 eligible cases. Of these, 2,626 (71.6%) were classified as NEGATIVE and 1,044 (28.4%) as POSITIVE. The class produced by the reconstructed predictor agreed with the class stored in the explanation file for every eligible case. The evaluated records belong to the cohort used during development of the underlying predictor. This is appropriate for analyzing explanation behavior over realistic activated-rule configurations, but it does not constitute an independent evaluation of predictive generalization. The predictive model itself is not retrained or selected in this experiment. Every method explained the class predicted for the individual case. SHAP used a 30-case background sampled from the cohort with a fixed random seed, along with 300 coalition samples. LIME used 1,000 neighborhood samples, did not discretize continuous attributes, and used the same fixed seed. For every case, four attribution vectors were constructed in the shared 79-attribute space: (i) the proposed TF-IDF vector; (ii) an unweighted local-rule vector containing attribute occurrence counts in R(x); (iii) absolute SHAP values for $\hat{y}(x)$; and (iv) absolute LIME coefficients for $\hat{y}(x)$. Absolute scores were normalized to sum to one before comparison. The unweighted local-rule vector serves as a structural baseline to examine the effect of TF-IDF reweighing, without changing granularity.^7^

Two complementary agreement measures were used. The Spearman rank correlation (Equation 13) measures global agreement across all 79 attributes. For attribution vectors a and b, it is defined as

$$\rho (a,b)=1-\frac{6\sum_{i=1}^{n}(r_{i}^{a}-r_{i}^{b}{)}^{2}}{n(n^{2}-1)},$$ (13)

where $r_{i}^{a}$ and $r_{i}^{b}$ are the ranks of attribute i and ties are handled using tie-adjusted ranks. Jaccard@5 (Equation 14) measures overlap between the two sets of highest-ranked attributes:

$$J@5(a,b)=\frac{\left|T_{5}^{a}\cap T_{5}^{b}\right|}{\left|T_{5}^{a}\cup T_{5}^{b}\right|},$$ (14)

where $T_{5}^{a}$ and $T_{5}^{b}$ contain the five most relevant attributes according to the respective methods. The Spearman correlation therefore captures similarity of the complete ranking, while Jaccard@5 focuses on the attributes most likely to be shown to an end user. Algorithm 2 summarizes the evaluation pipeline, where M is the set of explainable methods and C is the set of pairs of methods to be compared. Specifically, in the considered evaluation, M={TF−IDF,Frequency,SHAP,LIME}, and C={(TF−IDF,SHAP),(TF−IDF,LIME),(Frequency,SHAP),(Frequency,LIME)}.

Algorithm 2 **Attribution agreement comparison.**

**Require:** ⟨X,R,f,M,C⟩

**Ensure:** $\Gamma =\{\langle m_{i},m_{j},\rho ,J\rangle \}$

 1: Γ←∅                 ▹ Initialize agreement results

 2: **for** x∈X **do**

 3:  $c\leftarrow \arg\underset{j}{\max}f_{j}(x)$                    ▹ Get predicted class

 4:  $R_{x}\leftarrow \mathrm{rules}\;(x,R)$                    ▹ Extract rules activated by x

 5:  A←∅                       ▹ Initialize attribution assignment

 6:  **for** m∈M **do**

 7:   $\alpha_{m}\leftarrow \mathrm{attribution}\;(m,x,c,R_{x},f)$            ▹ Compute attribution according to m

 8:   $\alpha_{m}\leftarrow \mathrm{normalize}\;(\alpha_{m})$

 9:   $A\leftarrow A\cup \{\langle m,\alpha_{m}\rangle \}$

 10:  **end** **for**

 11:  **for** $\langle m_{i},m_{j}\rangle \in C$ **do**

 12:   $\rho \leftarrow \mathrm{spearman}\;(\alpha_{m_{i}},\alpha_{m_{j}})$                  ▹ Compute global ranking agreement

 13:   $J\leftarrow \mathrm{jaccard}@5\;(\alpha_{m_{i}},\alpha_{m_{j}})$                 ▹ Compute top-ranked attribute overlap

 14:   $\Gamma \leftarrow \Gamma \cup \{\langle m_{i},m_{j},\rho ,J\rangle \}$

 15:  **end** **for**

 16: **end** **for**

 17: Γ←summarize(Γ)                    ▹ Aggregate agreement measures over patients

18: **return** Γ

Table 2 summarizes the cohort-wide results. The TF-IDF-inspired method showed moderate global agreement with SHAP, with mean Spearman correlation 0.439, and lower agreement with LIME, with mean correlation 0.351. The same ordering was observed for the most highly ranked attributes: mean Jaccard@5 was 0.362 for TF-IDF vs. SHAP and 0.249 for TF-IDF vs. LIME. The 95% bootstrap confidence intervals for the respective means were [0.435,0.444], [0.348,0.354], [0.357,0.368], and [0.243,0.255]. The unweighted local-rule baseline exhibited slightly higher agreement with both post-hoc methods. Relative to local frequency, TF-IDF reduced mean Spearman agreement by 0.043 for SHAP and 0.028 for LIME, and reduced mean Jaccard@5 by approximately 0.029 for both methods. Patient-level bootstrap intervals for all four paired mean differences excluded zero. This modest reduction is consistent with the fact that the proposed method does not merely reproduce attribute occurrence counts. Rather, it reweights attributes according to their recurrence and distribution across activated rules. The result should therefore be interpreted as the measurable effect of the proposed weighting transformation, not as evidence that either SHAP or LIME provides a ground-truth ranking.

**Table 2:** Agreement of TF-IDF and the unweighted local-rule baseline with SHAP and LIME over 3,670 cases. Values are mean ± standard deviation.

Comparison	Spearman	Jaccard@5
TF-IDF vs SHAP	0.439±0.138	0.362±0.182
TF-IDF vs LIME	0.351±0.098	0.249±0.174
Local frequency vs SHAP	0.482±0.147	0.391±0.195
Local frequency vs LIME	0.378±0.089	0.278±0.162

Figure 4 shows the patient-level distributions. For TF-IDF vs. SHAP, the median Spearman correlation was 0.448, with an interquartile range of [0.353,0.539]. For TF-IDF vs. LIME, the median was 0.363, and the interquartile range was narrower, [0.289,0.415]. The corresponding median Jaccard@5 values were 0.429 and 0.250, respectively. Thus, TF-IDF more often shared a substantial portion of its five highest-ranked attributes with SHAP than with LIME, although variability across patients remained considerable.

**Figure 4:**
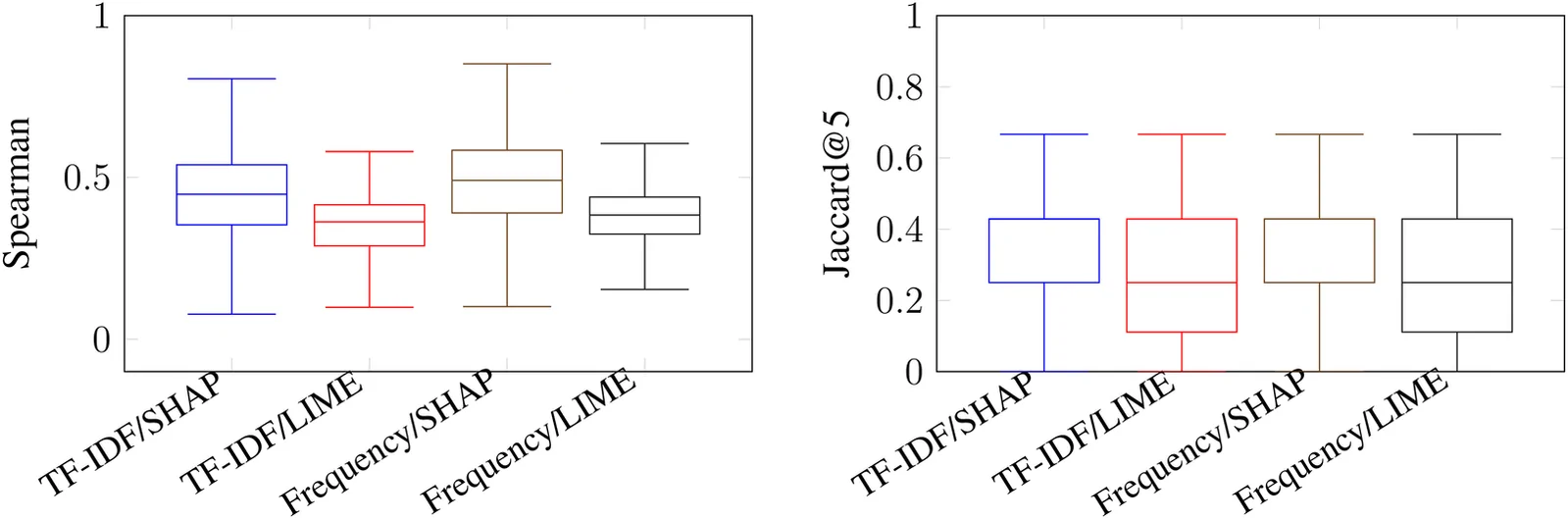
Distributions of Spearman correlation (left) and Jaccard@5 (right) over 3,670 patients. Boxes show the interquartile range and median.

The lower agreement with LIME can also be understood from the composition of its top-five sets. Four attributes (BMI, NODES_REMOVED, IMRT_UNKNOWN, and DIABETIES_UNKNOWN) appeared among LIME’s five highest-ranked attributes in more than 99% of cases. The fifth position varied more substantially. TF-IDF and SHAP produced more patient-dependent top-five compositions. This concentration explains why LIME can exhibit a relatively consistent global pattern while having limited overlap with case-specific TF-IDF rankings.

These results confirm that TF-IDF is more closely aligned with SHAP than with LIME, while also showing that the unweighted structural baseline retains somewhat greater agreement with both post-hoc methods. Agreement should be interpreted together with the faithfulness analysis in Section 4.3. The TF-IDF’s similarity to SHAP provides evidence of convergent attribution patterns, whereas the feature-replacement controls establish that its high-ranked attributes are behaviorally informative. Neither result implies clinical causality.

### 4.3 Analysis of faithfulness and stability

The agreement analysis in Section 4.2 evaluates whether different explanation methods produce similar rankings. The agreement does not establish whether the identified attributes influence the prediction function. To complement the analysis, this section performs: (i) a perturbation-based assessment of explanation faithfulness; (ii) an ablation against unweighted rule frequency; (iii) an assessment of LIME stability and local surrogate fidelity; and (iv) an analysis of the sensitivity of sampling-based SHAP to the background dataset. The analysis considered all eligible records from the PRE-ACT cohort used in the preceding comparison.

The sampling budgets of SHAP and LIME match those adopted in the agreement analysis described in the previous section. In addition, the current analysis repeats SHAP and LIME across five seeds, as repetitions are required to quantify their variability.^8^ To make the evaluation computationally feasible over the complete cohort, five independent runs were used for both LIME and SHAP. Each LIME run used 1,000 perturbed samples. Each SHAP run used 300 samples and a background of 30 cohort records independently selected for the corresponding seed. Faithfulness was assessed up to the ten highest-ranked attributes and was repeated with ten empirical reference records. The same fixed predictor, feature space, and patient cases were used for every explanation method.

Faithfulness was evaluated by testing whether replacing highly ranked attributes changes the prediction more than replacing arbitrary or low-ranked attributes. Four attribution rankings were considered: the proposed TF-IDF-inspired score, raw local-rule frequency, SHAP, and LIME. The raw-rule vector assigns each attribute its number of occurrences in the rules activated for the case and constitutes a direct ablation of the TF-IDF-inspired weighting at the same attribute granularity.

Let $c=\hat{y}(x)$ be the original predicted class for an input x and let $\pi_{m}(x)$ be the attribute ranking produced by method m. For a reference patient z, the perturbed input $x_{m,z}^{\left(k\right)}$ is obtained by replacing the first k attributes in $\pi_{m}(x)$ with their values in z. The probability drop after k replacements is measured according to Equation 15.

$$\Delta_{m,z}^{\left(k\right)}(x)=f_{c}(x)-f_{c}\;\left(x_{m,z}^{\left(k\right)}\right).$$ (15)

Then, Equation 16 computes the area over the perturbation curve up to ten attributes (AOPC@10) as:

$${\mathrm{AOPC}@10}_{m}(x)=\frac{1}{10\;|Z|}\sum_{z\in Z}\sum_{k=1}^{10}\Delta_{m,z}^{\left(k\right)}(x),$$ (16)

where Z contains ten reference patients sampled from the cohort. Higher AOPC indicates that attributes ranked near the top remove model support more rapidly.

Algorithm 3 summarizes the procedure, where M={TF−IDF,Frequency,SHAP,LIME} denotes the set of attribution methods, $M_{s}=\{\mathrm{SHAP},\mathrm{LIME}\}\subseteq M$ the subset evaluated through repeated runs, S the set of five experimental seeds, Z the set of empirical reference patients used for feature replacement, and C={Random,Bottom} the set of control rankings. For methods in $M_{s}=\{\mathrm{SHAP},\mathrm{LIME}\}$, multiple attribution vectors are generated using the experimental configurations described above. Pairwise agreement among these vectors quantifies repeated-run stability for LIME and sensitivity to the sampled background for SHAP. Feature replacement uses observed values from real patients instead of arbitrary constants. Nevertheless, combining attributes from different patients may produce an implausible clinical profile. Consequently, this experiment measures behavioral faithfulness to the implemented predictor and must not be interpreted as an estimate of clinical causation or the effect of a medical intervention. Results are summarized over patients using means, standard deviations, medians, and interquartile ranges. Confidence intervals for means and paired mean differences were obtained with 5,000 patient-level bootstrap resamples using a fixed random seed. Statistical inference is used to quantify the consistency of the observed differences over cohort cases, rather than to imply generalization to an independent clinical population.

Algorithm 3 **Faithfulness and stability evaluation.**

**Require:** $\langle X,R,f,M,M_{s},S,Z,C\rangle$

**Ensure:** Φ

 1: Φ←∅                    ▹ Initialize evaluation results

 2: **for** x∈X **do**

 3:  $c\leftarrow \arg\underset{j}{\max}f_{j}(x)$               ▹ Get predicted class

 4:  $R_{x}\leftarrow \mathrm{rules}\;(x,R)$                ▹ Extract rules activated by x

 5:  A←∅                   ▹ Initialize attribution assignment

 6:  **for** m∈M **do**

 7:   **if** $m\in M_{s}$ **then**

 8:    $A_{m}\leftarrow \emptyset$                     ▹ Initialize repeated attributions

 9:    **for** s∈S **do**

 10:     $\alpha_{m}^{s}\leftarrow \mathrm{attribution}\;(m,x,c,R_{x},f,s)$

 11:     $A_{m}\leftarrow A_{m}\cup \{\alpha_{m}^{s}\}$

 12:   **end** **for**

 13:   $\sigma_{m}\leftarrow \mathrm{stability}\;(A_{m})$                 ▹ Evaluate variability across runs

 14:   $\alpha_{m}\leftarrow \mathrm{average}\;(A_{m})$                 ▹ Aggregate repeated attributions

 15:   $\Phi \leftarrow \Phi \cup \{\langle x,m,\sigma_{m}\rangle \}$

 16:  **else**

 17:   $\alpha_{m}\leftarrow \mathrm{attribution}\;(m,x,c,R_{x},f)$

 18:   **end** **if**

 19:   $\alpha_{m}\leftarrow \mathrm{normalize}\;(\alpha_{m})$

 20:   $A\leftarrow A\cup \{\langle m,\alpha_{m}\rangle \}$

 21:  **end** **for**

 22:  $E_{x}\leftarrow \mathrm{featureEffect}\;(x,f,c,Z)$                ▹ Estimate empirical single feature effects

 23:  **for** m∈M∪C **do**

 24:   $\pi_{m}\leftarrow \mathrm{ranking}\;(m,A)$                   ▹ Generate attribution or control ranking

 25:   $\Delta_{m}\leftarrow \emptyset$

 26:   **for** z∈Z **do**

 27:    **for** k=1,…,10 **do**

 28:     $x_{m,z}^{\left(k\right)}\leftarrow \mathrm{replace}\;(x,\pi_{m},z,k)$                  ▹ Replace first *k* ranked attributes

 29:     $\Delta_{m,z}^{\left(k\right)}\leftarrow f_{c}(x)-f_{c}(x_{m,z}^{\left(k\right)})$

 30:     $\Delta_{m}\leftarrow \Delta_{m}\cup \{\Delta_{m,z}^{\left(k\right)}\}$

 31:   **end** **for**

 32:  **end** **for**

 33:  $\phi_{m}\leftarrow \mathrm{faithfulness}\;(\Delta_{m},E_{x})$                ▹ Compute faithfulness measures

 34:  $\Phi \leftarrow \Phi \cup \{\langle x,m,\phi_{m}\rangle \}$

 35:  **end** **for**

 36: **end** **for**

 37: Φ←summarize(Φ)         ▹ Aggregate measures over patients

 38: **return** Φ

Table 3 reports the perturbation results. SHAP produced the largest mean AOPC@10 (0.378), followed by raw rule frequency (0.299), TF-IDF-inspired (0.259), and LIME (0.225). All four explanation methods substantially exceeded random ordering (0.039) and the bottom-TF-IDF control (0.025). In particular, the paired mean AOPC difference between TF-IDF-inspired and random ordering was 0.219 (95% bootstrap confidence interval [0.214,0.225]).

**Table 3:** Faithfulness results over 3,670 cases. The faithfulness correlation is Spearman correlation with empirical single-feature effects. It is not defined for the random and bottom-ranked controls because these do not provide an attribution vector intended to explain the prediction.

Ranking	AOPC@10	Median [IQR]	Class-change rate	Faithfulness ρ
SHAP	0.378±0.232	0.351[0.200,0.535]	0.350±0.275	0.336±0.214
Raw rule frequency	0.299±0.216	0.278[0.121,0.443]	0.278±0.263	0.169±0.198
TF-IDF	0.259±0.222	0.215[0.065,0.395]	0.244±0.266	0.153±0.209
LIME	0.225±0.216	0.140[0.060,0.352]	0.207±0.258	0.251±0.149
Random	0.039±0.067	0.005[0.000,0.051]	0.039±0.083	–
Bottom TF-IDF	0.025±0.061	0.000[0.000,0.017]	0.021±0.072	–

The class-change results show the same ordering. Replacing the highest-ranked TF-IDF-inspired attributes changed the predicted class in 24.4% of the evaluated deletion steps, compared with 3.9% under random ordering and 2.1% for bottom-ranked TF-IDF-inspired attributes. This large separation between the top and bottom of the TF-IDF-inspired ranking supports the conclusion that the proposed score concentrates on attributes that are behaviorally relevant to the implemented predictor. The ablation against raw frequency provides a more specific assessment of the weighting scheme. The raw frequency exceeded the TF-IDF-inspired by a paired mean AOPC of 0.040 (95% bootstrap confidence interval [0.036,0.044]). Its mean correlation with single-feature effects was also slightly higher (0.169 vs. 0.153). Thus, the TF-IDF-inspired transformation remains substantially more faithful than random and bottom-ranked controls, but it does not improve perturbation faithfulness over the unweighted structural baseline. This is an informative trade-off rather than evidence that the score is unfaithful. The TF-IDF-inspired method is designed to reorganize recurrent rule evidence into a compact relevance representation. In contrast, raw frequency more directly preserves the multiplicity of attribute occurrences in the activated rules. Accordingly, the added value of weighting should be attributed to its relevance organization and presentation properties, not to an improvement over raw frequency on these perturbation metrics.

SHAP’s advantage is expected because its objective is explicitly defined through changes in model output under feature perturbations. As shown in Figure 5, its AOPC exceeded TF-IDF by 0.119 on average (95% bootstrap confidence interval [0.114,0.124]). Nevertheless, TF-IDF-inspired exceeded LIME by 0.034 (95% bootstrap confidence interval [0.029,0.039]). The single-feature correlations provide a complementary view. SHAP obtained the highest mean correlation (0.336), followed by LIME (0.251), raw frequency (0.169), and TF-IDF-inspired (0.153). These modest correlations also reflect the redundant structure of the rule-based model since changing one condition may have little effect when other activated rules support the same class, and multiple removals can deactivate several overlapping paths.

**Figure 5:**
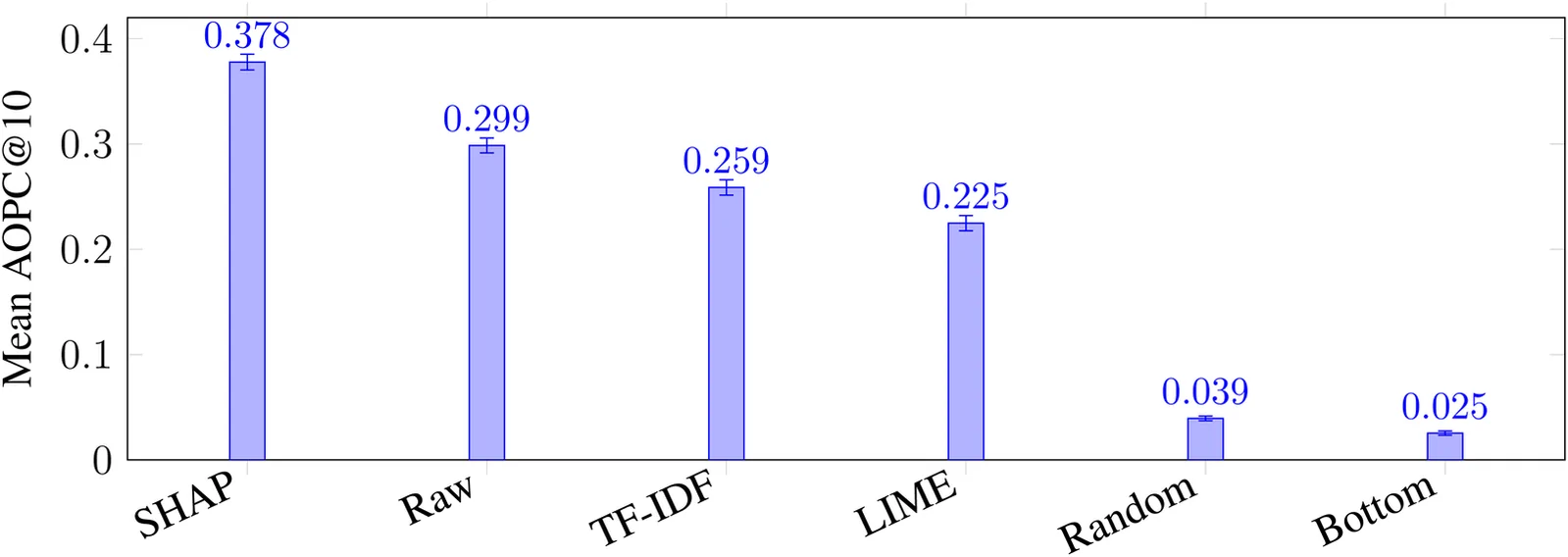
Mean AOPC@10 across cases. Error bars show 95% bootstrap confidence intervals for the mean; “Raw” denotes unweighted local-rule frequency and “Bottom” the lowest-ranked TF-IDF-inspired attributes.

Stability was quantified across the (52)=10 pairs of repeated explanations for each patient. Spearman correlation measures global rank consistency, while Jaccard@10 measures overlap among the ten highest-ranked attributes. For LIME, local surrogate fidelity was assessed using the weighted coefficient of determination ($R^{2}$) returned by the local regression and the absolute difference between the surrogate’s local prediction and $f_{c}(x)$. For SHAP, the same pairwise stability metrics quantify sensitivity to independently sampled background datasets.

The results are reported in Table 4. LIME exhibited moderate-to-high consistency with the pairwise Spearman correlation 0.619 (95% bootstrap confidence interval [0.618,0.621]) and the mean Jaccard@10 0.671 ([0.669,0.672]). The mean local surrogate $R^{2}$ was 0.560 ([0.559,0.561]), while the mean absolute local prediction error was 0.187 ([0.181,0.193]). These results indicate that LIME’s repeated explanations share a substantial subset of their top attributes, but its linear surrogate only partially approximates the locally discontinuous rule-based prediction function.

**Table 4:** LIME stability and fidelity, and SHAP sensitivity to the background dataset, over 3,670 cases. Pairwise metrics are averaged over the ten pairs formed by five runs.

Measure	Mean ± SD	Median	IQR
LIME pairwise Spearman	0.619±0.039	0.604	[0.595,0.636]
LIME Jaccard@10	0.671±0.048	0.684	[0.646,0.697]
LIME local surrogate $R^{2}$	0.560±0.041	0.554	[0.551,0.557]
LIME local absolute error	0.187±0.178	0.120	[0.053,0.267]
SHAP background Spearman	0.592±0.085	0.588	[0.535,0.650]
SHAP background Jaccard@10	0.433±0.083	0.423	[0.374,0.484]

SHAP showed material sensitivity to the choice of background data. Across independently sampled 30-case backgrounds, mean pairwise rank correlation was 0.592 (95% bootstrap confidence interval [0.590,0.595]), and mean Jaccard@10 was 0.433 ([0.430,0.435]). Thus, fewer than half of the distinct attributes appearing in two top-ten sets overlapped on average. This variability should be considered when interpreting a single sampling-based SHAP explanation and also helps contextualize imperfect cross-method agreement. In contrast, the TF-IDF-inspired and raw-frequency explanations are deterministic for a fixed input and fixed rule set. This deterministic property should be distinguished from robustness to changes in the patient input, which was not assessed here.

In conclusion, this analysis distinguishes three notions whose differences would otherwise remain blurred when considering agreement alone. First, the proposed TF-IDF-inspired ranking is behaviorally informative because perturbing its top attributes affects the predictor substantially more than random or bottom-ranked controls, and more closely to the raw model. Second, the raw-frequency ablation is somewhat more perturbation-faithful, clarifying that TF-IDF-inspired weighting introduces a measurable trade-off rather than uniformly improving every explanation property. Third, LIME and sampling-based SHAP exhibit non-negligible dependence on random sampling and, for SHAP, the reference background. These findings show that the TF-IDF-inspired method provides deterministic, computationally efficient explanations grounded in activated rules and identifies features that are substantially more influential than random or bottom-ranked controls. Although its perturbation faithfulness is lower than that of SHAP and raw rule frequency, it offers a structure-aware relevance representation without sampling or surrogate models. Cross-method agreement should therefore be interpreted as complementary evidence of consistency, not as proof of explanation correctness or clinical causality.

### 4.4 User study of perceived usefulness and interpretability

To understand whether the proposed analysis and the computed relevance scores can help users interpret the output, we designed a user study where participants were asked to evaluate the XAI model’s output presented through three different representations:

- *Raw output containing propositional rules*, as generated by the model, consisting of the estimated risk with its confidence interval, accompanied by locally activated rules representing the attributes contributing to the risk.
- *Radar graph displaying the contributing factors*, namely the processed information using the technique presented in this paper, visualized through a radar chart where the contributing factors to the risk are represented as the spikes, and their weight is conveyed through the chart.
- *List of factors contributing to the risk prediction*, namely the processed information using the technique from this work, presented as a list of contributing variables where their impact on the risk is represented as a percentage.

The objective of the user study is intentionally different from that of the quantitative attribution analysis presented in the previous sections. While the former evaluates properties of the TF-IDF-inspired weighting mechanism, the user study evaluates the complete explanation pipeline as experienced by end users, including relevance weighting, semantic aggregation, and visual presentation. Accordingly, the study is not intended to isolate the independent contribution of TF-IDF-inspired weighting, semantic abstraction, or visualization, but rather to assess whether their integration produces explanations that users perceive as understandable and useful in realistic interaction scenarios.

The three output visualizations (Figure 6) were generated for four profiles representing realistic patients with varying risk levels. Participants in the study were asked to indicate, on a 5-point Likert scale, the level of *interpretability* and *usefulness* of the information conveyed in each output representation across all four patient profiles. To estimate a valuable sample size, an a priori power analysis was conducted using G*Power (42). To detect an effect size of 0.6 with 80% power and a significance level of 0.05, a sample of 28 participants was required. More than 28 participants were involved in order to mitigate the risk of sample size reduction due to subject exclusion during the analysis. Specifically, 30 participants were recruited via convenience sampling in this evaluation phase, including both AI experts and non-AI experts. One participant was excluded from the statistical analysis due to incomplete responses. Therefore, the final analysis included 29 participants. No further information on the participants was collected, in accordance with the data minimization principle of GDPR, Article 5(1)(c) (43).

**Figure 6:**
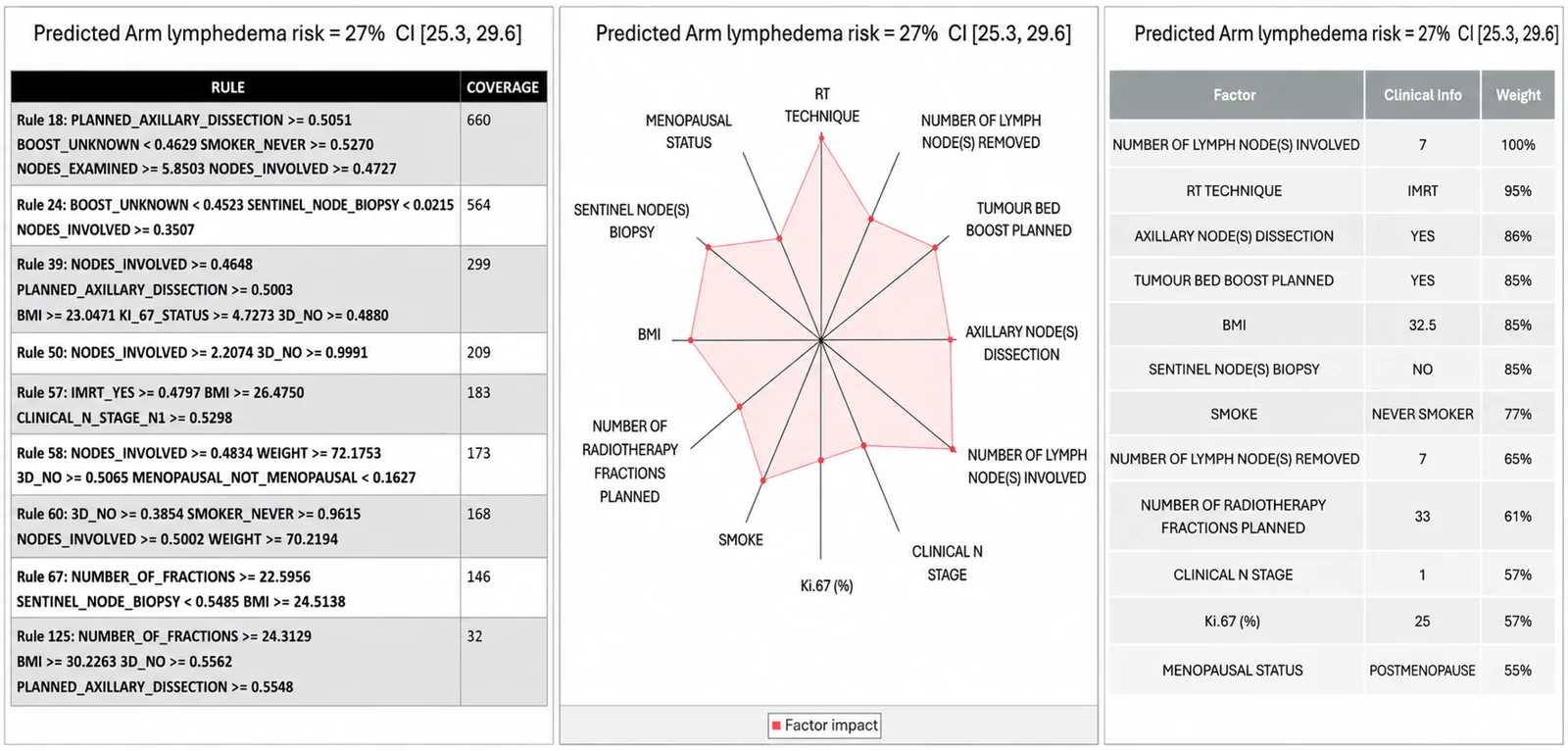
The three different output visualizations for a single patient profile. From left to right: Raw output containing propositional rules; Radar graph displaying the contributing factors; List of factors contributing to the risk prediction.

Figure 7 shows statistically significant differences in the scores of both *Interpretability* and *Usefulness* for the three visualization options. A repeated measures ANOVA revealed a significant main effect of the different types of visualization on *Interpretability*, ($F_{\left(2,56\right)}=35.9$; p<0.001, $\eta_{G}^{2}$ = 0.375). Post-hoc analysis showed no significant differences between the radar graph and the list of factors. Interestingly, the interpretability scores of rule-based outputs were significantly lower than scores of the list ($t_{\left(28\right)}=7.74$; p<.0001) and radar visualization ($t_{\left(28\right)}=7.52$; p<0.001) The same results emerged for the *Usefulness* ($F_{\left(2,56\right)}=20.14$; p<0.001, $\eta_{G}^{2}$ = 0.235), and post-hoc analysis found significant differences between rule-based output usefulness compared to both the graphical visualization ($t_{\left(28\right)}=4.59$; p<0.001) and the visualization through the list of contributing factors ($t_{\left(28\right)}=5.77$; p<0.001). Finally, comparing the assessment scores of AI experts and non-AI experts, no significant differences were found in the judgments regarding the *Interpretability* and *Usefulness* of the three modalities.

**Figure 7:**
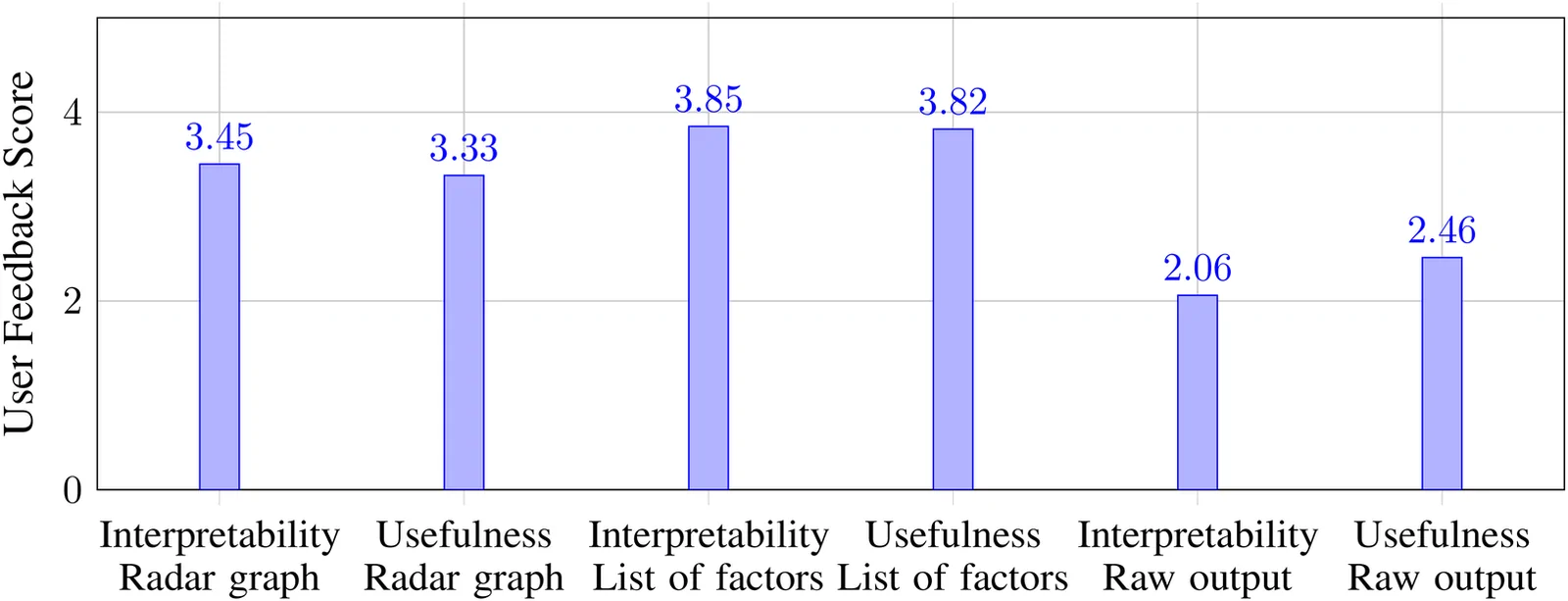
The mean scores of both *Interpretability* and *Usefulness* of the three visualization options.

## 5 Discussion

The results indicate that explainability in clinical decision-support systems depends not only on whether the predictive model exposes its internal logic, but also on how that logic is organized and communicated. Although the rules of a symbolic predictor can be inspected directly, a local prediction may activate several rules containing numerous low-level attributes. Presenting this information through clinically meaningful semantic factors reduces the user-facing explanation space from 79 model attributes to 29 clinical factors, corresponding to an approximate 63% reduction in dimensionality. The user study further supports the perceived interpretability and usefulness of the integrated explanation framework, including semantic abstraction and visualization.

The user study reported in Section 4.4 compared raw propositional rules, a radar representation of the weighted factors, and a ranked list of the same factors over four realistic patient profiles. Among the 29 participants included in the analysis, both processed representations received higher ratings than the raw-rule output. Mean interpretability was 3.45 for the radar and 3.85 for the list, compared with 2.06 for raw rules; mean usefulness was 3.33 and 3.82, respectively, compared with 2.46 for raw rules. The effect of representation was significant for both interpretability and usefulness (p<0.001), while no significant interpretability difference was found between the radar and list. Moreover, no significant differences were observed between participants with and without AI expertise. These findings show that the advantage over raw rules was observed with both processed formats and suggest that organizing rule evidence into a concise factor-level representation can assist both technical and non-technical users. The cross-method comparison provides evidence of convergent validity rather than a ground truth for explanation quality. Over the complete eligible cohort, the proposed method continues to show greater agreement with SHAP than with LIME in both global feature ordering and top-feature overlap, consistently with the results reported in Section 4.2. This indicates that the structure-aware representation preserves a meaningful part of the attribution patterns identified by established post-hoc techniques. Nevertheless, neither SHAP nor LIME constitutes a reference explanation whose agreement alone can establish correctness. This consideration motivated the complementary faithfulness and stability evaluation in Section 4.3. The feature-replacement analysis shows that the proposed ranking is behaviorally informative with respect to the reconstructed rule-based predictor. Replacing the ten highest-ranked TF-IDF-inspired attributes produced a mean AOPC@10 of 0.259, compared with 0.039 for random ordering and 0.025 for the lowest-ranked TF-IDF-inspired attributes. The predicted class changed in 24.4% of the TF-IDF-directed perturbation steps, but in only 3.9% and 2.1% of the random and bottom-ranked controls, respectively. The clear separation from both controls provides evidence that attributes receiving high TF-IDF-inspired scores are materially related to the behavior of the implemented predictor. This result strengthens the evaluation beyond cross-method agreement, while remaining a statement about model behavior rather than clinical causation. The comparison with unweighted local-rule frequency constitutes an ablation of the proposed weighting scheme at the same attribute granularity. Raw frequency obtained a higher mean AOPC@10 than TF-IDF-inspired (0.299 vs. 0.259) and a slightly higher correlation with empirical single-feature effects (0.169 vs. 0.153). Therefore, the TF-IDF-inspired transformation does not improve perturbation faithfulness over the direct frequency baseline. Instead, it introduces a measurable trade-off: it reorganizes evidence according to its recurrence and distribution across activated rules, but this re-weighting can attenuate the direct relationship between occurrence counts and the effect of removing features. The added value of the proposed scheme should consequently not be presented as uniformly superior faithfulness. Its contribution lies in providing a deterministic and computationally lightweight relevance representation that supports the semantic organization of complex rule evidence, while retaining faithfulness substantially above the control conditions.

The technical and user-centered results are complementary in this respect. The perturbation analysis shows that direct rule frequency retains more of the predictor’s removal sensitivity, whereas the user study shows that exposing the original rules is less understandable and useful than presenting the processed factor-level explanation. These findings should not be combined into a causal claim that TF-IDF-inspired weighting itself improves user performance, since semantic grouping and presentation also changed between conditions. They instead demonstrate that optimizing solely for perturbation faithfulness does not address the complete communication problem: a clinically useful explanation must preserve a defensible connection to model behavior while presenting that information at a level users can understand. The proposed framework occupies this intermediate position, retaining faithfulness well above the controls while supporting the more accessible representations evaluated in the user study. Furthermore, the proposed framework is independent of the training procedure of the underlying predictor and can in principle be applied to any rule-based model exposing its activated rules and associated variables. Its main methodological contribution is therefore a lightweight mechanism for transforming complex rule activations into a ranked, semantically organized explanation without additional model evaluations at explanation time.

The empirical results identify a three-way trade-off. SHAP is most aligned with perturbation faithfulness but is computationally demanding and background-dependent; raw frequency most directly preserves the structural multiplicity of activated conditions; and TF-IDF-inspired provides a compact, deterministic weighting of that structural evidence, with lower perturbation faithfulness than SHAP and raw frequency but substantially higher faithfulness than random and bottom-ranked controls. Taken together, these results indicate that the contribution of the proposed framework should not be interpreted as an improvement of perturbation faithfulness over direct rule frequency. Rather, the framework introduces a deterministic mechanism for organizing structural rule evidence into a semantically meaningful relevance representation that can subsequently be communicated through user-oriented explanations.

## 6 Conclusions and future work

This work presented a lightweight, structure-aware explanation method for rule-based predictors. By representing activated rules as a document-like structure and applying a TF-IDF-inspired weighting scheme, the method produces a ranked representation of the attributes supporting an individual prediction without requiring input perturbations, surrogate models, repeated predictor evaluations, or access to the original training data at explanation time. Combined with semantic aggregation and tailored presentation, this representation is intended to make complex rule-based explanations more manageable in clinical communication settings.

The expanded evaluation separates properties that cannot be inferred from one another. The cohort-wide comparison with SHAP and LIME assesses cross-method agreement and indicates that the proposed approach preserves a meaningful part of established attribution patterns, particularly those produced by SHAP. The new feature-replacement analysis directly assesses behavioral faithfulness: TF-IDF-inspired obtained a mean AOPC@10 of 0.259, markedly above random ordering (0.039) and bottom-ranked attributes (0.025). At the same time, SHAP (0.378) and raw rule frequency (0.299) achieved higher AOPC values. The raw-frequency ablation therefore shows that TF-IDF-inspired weighting does not increase perturbation faithfulness over the unweighted structural baseline, but retains a substantial relationship with model behavior while providing a deterministic relevance representation. The stability analysis further shows that LIME varies across random seeds and only partially approximates the local rule function, while sampling-based SHAP is sensitive to its background dataset. These findings support the proposed approach as an efficient structural explanation, but not as a replacement for perturbation-based analysis when counterfactual sensitivity is the primary objective. The user-centered findings complete this assessment by addressing a different dimension of explanation quality. Across four patient profiles, 29 participants rated the radar and list representations significantly more interpretable and useful than the original propositional rules, and this pattern did not differ between AI experts and non-experts. Together with the technical results, this supports the framework’s intended role as a bridge between model-level rule evidence and end-user communication: it remains connected to the predictor’s behavior, is deterministic and inexpensive to compute, and enables concise representations that users rated more favorably than raw rules. Because the user study evaluated the integrated pipeline, this conclusion applies to the combined semantic aggregation, weighting, and presentation rather than to TF-IDF-inspired weighting in isolation. The technical and user-centered evaluations therefore support different aspects of the proposed framework: the former characterizes the structural and behavioral properties of the weighting mechanism, whereas the latter assesses the perceived quality of the complete explanation delivered to end users.

Several limitations should guide the interpretation of these results. The evaluation concerns a single breast-cancer risk predictor, and the assessed cohort was involved in developing the underlying model; generalization must therefore be established on independent data, other rule-based architectures, and additional clinical domains. The reconstructed predictor returned saturated probability-like scores for the evaluated cases, and its rule-confidence output was not assessed as a calibrated clinical probability. Moreover, empirical replacement can create combinations of attributes that are uncommon or clinically implausible. The reported AOPC and class-change measures consequently quantify faithfulness to the implemented predictor rather than causal effects or expected changes in patient outcomes. The semantic taxonomy was developed through iterative co-design involving AI researchers and clinical experts, but it was not validated through an independent annotation study or inter-rater agreement analysis. In addition, the existing user study evaluates the complete user-facing framework and does not independently isolate raw frequency, TF-IDF-inspired weighting, semantic grouping, and visualization. Its findings therefore support the interpretability of the integrated presentation rather than a specific user-performance advantage caused by TF-IDF-inspired weighting alone. A factorial user study holding visualization and semantic grouping constant while varying only the weighting scheme would be required to establish that contribution independently.

Future work will address these limitations in four directions. First, the framework and its faithfulness should be evaluated on independent cohorts, additional rule-based predictors, and other application domains. Second, alternative information-retrieval weighting schemes should be examined against raw-frequency baselines to determine whether semantic organization can be improved without the observed loss in perturbation faithfulness. Third, robustness should be evaluated under clinically plausible local changes using conditional or constraint-aware perturbations, thereby avoiding unrealistic combinations of patient attributes and distinguishing fixed-input determinism from neighborhood stability. Finally, the semantic taxonomy and its individual presentation components should be validated through independent expert assessment and controlled user studies. Together, these extensions would provide a more complete account of structural fidelity, behavioral faithfulness, robustness, and human usefulness while preserving the computational advantages of the proposed method.

## Data Availability

The data analyzed in this study is subject to the following licenses/restrictions: Data is confidential and cannot be shared. Requests to access these datasets should be directed to the corresponding author, and the code can be found at the following link: https://doi.org/10.5281/zenodo.20813879.

## Footnotes

^1^ https://preact-horizoneurope.eu/

^2^ https://preact-horizoneurope.eu/

^3^ https://github.com/Jean-Marc-B/dimlpfidex_Hepia

^4^Software library available on https://github.com/Jean-Marc-B/dimlpfidex_Hepia

^5^Shareable data and code of the algorithms are available on Zenodo (39), doi: https://doi.org/10.5281/zenodo.20813879

^6^The radar charts show the interpretation of 9 selected profiles with an average of 7 activated rules and diverse attribute sets.

^7^The unweighted local-rule frequency baseline is obtained by counting the occurrences of each attribute in the set of activated rules for a patient, without applying the proposed TF-IDF weighting.

^8^The agreement experiment used a single fixed configuration per patient so that every comparison was made under identical conditions.
